# Association between Parents’ Relationship, Emotion-Regulation Strategies, and Psychotic-like Experiences in Adolescents

**DOI:** 10.3390/children9060815

**Published:** 2022-05-31

**Authors:** Chenyu Zhan, Ziyu Mao, Xudong Zhao, Jingyu Shi

**Affiliations:** 1 Shanghai East Hospital, Tongji University School of Medicine, Shanghai 200120, China; 1710447@tongji.edu.cn; 2 Ruijin Hospital Luwan Branch, Shanghai JiaoTong University School of Medicine, Shanghai 200011, China; ziyum7@sina.com; 3 Clinical Research Center for Mental Disorders, Chinese-German Institute of Mental Health, Shanghai Pudong New Area Mental Health Center, School of Medicine, Tongji University, Shanghai 200124, China; 4 Division of Medical Humanities & Behavioral Sciences, Tongji University School of Medicine, Shanghai 200331, China; shijingyu2005@126.com

**Keywords:** psychotic-like experiences, emotion regulation, parents’ relationship, adolescent

## Abstract

This study aimed to examine the association between the psychotic-like experiences (PLEs) and emotion-regulation (ER) strategies of adolescents and their parents’ relationship, and we hypothesized that the parents’ relationship moderates the link between ER strategies and PLEs. We recruited a total of 2708 first-year college students (1659 males and 1049 females) aged 15–20 years (mean = 17.9). Participants completed assessments of PLEs, their use of ER strategies, and reported their parents’ relationship as harmonious, conflicting, or divorced. Regression analyses indicated that the lower the use of the emotion-reappraisal strategy, the greater the use of the emotion-suppression strategy and that parental conflict or divorce predicted the number of PLEs endorsed and the level of distress from the PLEs. The parents’ relationship moderated the association between ER strategies and distress from PLEs. Among those who reported parental conflict or divorce, their lower use of the reappraisal strategy predicted their experiencing higher levels of distress from their PLEs. This study suggested the direct and interactive influence of the parents’ relationship and ER strategies on the presence of PLEs and PLE-related distress levels among adolescents, which may represent potential intervention targets.

## 1. Introduction

Psychotic-like experiences (PLEs) are characterized by the presence of subthreshold psychotic symptoms and are frequently reported in the general population, with a lifetime prevalence of 5.8% [1]. Adolescents have more PLEs than adults. Although these PLEs are mild and transient for most people, many studies suggest that PLEs are associated with elevated risk for not only psychotic [2] and non-psychotic disorders [3] but also for a multitude of adverse mental health outcomes, including cognitive, social, and role impairment [4], increased help-seeking [5] and self-injury [6], and poor health-related quality of life [7] as well as affective dysregulation [8].

Adolescence is a critical period for developing emotional regulation (ER). According to the process model formulated by Gross et al., cognitive reappraisal (e.g., the reinterpretation of an emotional situation) and expressive suppression (e.g., the inhibition of the expression of emotions) are two widely studied common emotion-regulation strategies [9]. Cognitive reappraisal is considered to be an adaptive ER strategy that is beneficial for mental health, while expressive suppression is more maladaptive and associated with psychopathology [10]. ER impairments exist across the continuum of psychosis and may constitute a risk factor for developing psychotic symptoms. A recent meta-analysis showed that individuals with psychosis are more likely to use suppression than reappraisal [11]. The same pattern was also observed in people with PLEs and at clinical high risk for psychosis [12,13]. Several studies have suggested that the greater use of suppression could be a risk factor for PLEs and is associated with increased subclinical positive symptoms [14], negative symptoms [15], and contributes to the development of the need for clinical care [16]. Mixed findings showed that reappraisal might be a protective factor against increased distress related to PLEs [15], while others found no or the opposite effect for subclinical psychotic symptoms [17,18]. The link between the use of ER strategies and PLEs among adolescents remains unclear.

Adverse social and environmental factors in childhood and adolescence, including stressful and traumatic life events such as bully victimization [19] and child abuse [20], are linked to increased susceptibility to PLEs. Especially during the COVID-19 pandemic, the stress caused by the lockdown has led to increased PLEs among adolescents [21], and positive family functioning can be protective during this crisis [22]. Among these factors, family-related factors have received much concern in the study of psychosis. Conflicts in the family become more frequent and intense during adolescence, which may have a significant influence on children’s development and psychopathology. Parental conflicts can affect parents’ interactions with children and their parenting behavior and have a direct effect on children’s psychological distress [23]. Some studies have suggested that exposure to parental conflict or divorce may be related to depression, anxiety, dissociation, substance abuse, worsening health conditions, and poor functioning among people with psychosis [24,25,26]. In turn, positive family function may serve as a protective factor against the deleterious effects of attenuated psychotic symptoms on social functioning for individuals prone to psychosis [27] and reduce the relapse rates of patients with schizophrenia [28]. Adolescents are susceptible to family influence. Negative parental relationships, particularly parental conflict or divorce, remain a vital contributor to multiple poor outcomes among adolescents, including emotional, behavioral, social, and academic problems [29] and have long-term adverse effects on mental health [30]. The association between the parents’ relationship and PLEs is still unclear among adolescents.

Researchers also have recognized the importance of family context in shaping emotion regulation [31,32]. The parents’ relationship can impact adolescents’ emotional development. Children’s awareness of parental conflict and their coping responses toward the conflict may play a key role in how parental conflict affects children’s adjustment [23]. Even if the conflict is not directed at the child, children exposed to parental conflict may still exhibit less adaptive ER strategies [33]. Positive coping responses, such as adjusting to conflict situations through cognitive reappraisal, can prevent the externalizing of problems in the context of marital conflict [34]. Evidence indicates that positive family relationships may predict greater emotional reappraisal, whereas family conflict predicts more emotional suppression in young adults [35]. However, whether the interaction between ER strategies and the parents’ relationship is related to PLEs remains unknown.

This study aimed to investigate the association between the parents’ relationship, ER strategies, and PLEs and to further study whether the parents’ relationship is a possible moderator of the link between ER strategies and PLEs in non-clinical adolescents. We hypothesized the number of PLEs endorsed and the distress from the PLEs to be positively associated with suppression and parental conflict or divorce and negatively associated with reappraisal and positive parent relationships. Furthermore, the relationship between ER strategies and PLEs is moderated by the parents’ relationship. A positive parents’ relationship weakens the adverse influence of the maladaptive pattern of ER strategies on PLEs, while parental conflict or divorce exacerbates it.

## 2. Materials and Methods

### 2.1. Study Design and Participants

This study was a cross-sectional survey conducted from September 2016 to December 2016 in Tongji University. The questionnaires were distributed to freshmen attending the mental health education course. Of the 3147 students invited to participate in the survey, 3120 returned the questionnaires. In total, a sample of 2708 college students (1659 males and 1049 females) aged 15–20 years (mean = 17.9, SD = 0.7) were recruited after excluding incomplete answers, giving a response rate of 86.1%. There were no significant differences between those who did not complete the scales and the responders in terms of gender and age (*p* > 0.05). All participants provided written informed consent prior to the study, and participants under the age of 18 were given written consent by their parents. The questionnaires were completed in a quiet classroom environment and took about 15–25 min. This study was approved by the ethics committee of Tongji University School of Medicine on 26 February 2016.

### 2.2. Measurements

#### 2.2.1. Psychotic-like Experiences

The 16-item version of the Prodromal Questionnaire (PQ-16) [36] is a self-report questionnaire screening for the presence of PLEs and associated distress. PQ-16 comprises nine items on perceptual abnormalities and hallucinations; five items on unusual thought content, paranoia, and delusional ideas; and two items on negative symptoms. Responses are scored as “yes” or “no” for each item. If participants endorsed an item, they were also asked to rate the severity of the distress using a 4-point Likert-type scale from 0 (no) to 3 (severe). The psychometric properties of the Chinese version of the PQ-16 have been well established (Cronbach’s α = 0.72) [37]. A total distress score threshold of ≥9 was recommended in a non-help-seeking population, with 85% sensitivity and 87% specificity [37]. In this study, the Cronbach’s α coefficient was 0.79 for the PQ-16 items endorsed and α= 0.82 for the distress of PQ-16.

#### 2.2.2. Emotion-Regulation Strategies

The habitual use of the emotion-regulation strategies was assessed using the Emotion Regulation Questionnaire (ERQ) [38]. It consists of six items examining reappraisal and four items measuring suppression, scored on a 7-point Likert-type scale from 1 (strongly disagree) to 7 (strongly agree). Higher scores on each scale indicate a greater tendency to use the corresponding strategy. The Chinese version of the ERQ has satisfactory psychometric properties. The reliability estimates were good among college students, with Cronbach’s α = 0.783 for the cognitive reappraisal scale and Cronbach’s α = 0.772 for the expressive suppression scale [39]. In this study, the Cronbach’s α coefficient was 0.80 for reappraisal and α = 0.71 for suppression.

#### 2.2.3. The Parents’ Relationship

We assessed the parents’ relationship by asking participants how their parents got along in the prior 12 months, with responses dichotomized into: (i) harmonious and (ii) conflicting or divorced.

#### 2.2.4. Sociodemographic Characteristics

The controlled sociodemographic variables included gender, age, single-child status (yes or no), and monthly family income, which were categorized as < CNY 2000 (Chinese yuan), CNY 2000–4999, and ≥ CNY 5000.

### 2.3. Statistical Analysis

Descriptive analysis was conducted to describe the participants’ characteristics and measures of ER strategies and PLEs. Hierarchical linear regression analyses were performed to investigate the direct and moderating effects of the parents’ relationship in the association between ER strategies and PLEs. The two regression models were assessed separately with the dependent variables (a) the number of PLEs endorsed and (b) the distress of PLEs. All variables were mean-centered and tested for normal distribution by the Shapiro–Wilk test. Because all the PQ-16 scores were skewed, we log-transformed them before being centered. Categorical variables were included as dummy variables. In step one of each model, age, gender, and single-child status were entered as covariates. In step two, the parents’ relationship and the subscales for reappraisal and suppression from the ERQ were entered. In step three, the interaction terms for the parents’ relationship and the ERQ subscales were entered into the model separately. Then, simple slope analyses were conducted to explore the moderation effects. Two-tailed tests were used with a significance level set at 0.05. Analyses were performed with SPSS version 19.0 for Windows.

## 3. Results

### 3.1. Sociodemographic Characteristics and Psychological Measures

Table 1 displays the descriptive statistics for sociodemographic and psychological measures. Of the 2708 participants, 89.2% endorsed at least one item on the PQ-16, 86.8% endorsed at least one positive symptom, and 54.1% endorsed at least one negative symptom. A percentage of 2.8% (*n* = 75) scored above the distress score cutoff of ≥9, indicating a higher risk for proneness to psychosis.

### 3.2. The Number of Psychotic-like Experiences Endorsed

Hierarchical multiple regression analyses investigating the association between emotion-regulation strategies, parents’ relationship, and the number of PLEs endorsed are presented in Table 2. These models were adjusted for age, gender, and single-child status. Regression models indicated that lower use of reappraisal, greater use of suppression, and parental conflict or divorce had significant associations with the number of PLEs endorsed (R^2^ = 0.123, *p* < 0.001). The parents’ relationship did not moderate the associations between emotion-regulation strategies and the number of PLEs endorsed.

### 3.3. Distress Related to Psychotic-like Experiences

Table 2 also showed that emotion-regulation strategies and the parents’ relationship are associated with the distress from PLEs (R^2^ = 0.138, *p* < 0.001). After controlling for covariates, lower use of reappraisal, greater use of suppression, and parental conflict or divorce significantly predicted higher distress from PLEs. There were significant interaction effects for parents’ relationship and reappraisal on the distress from PLEs.

**Figure 1:**
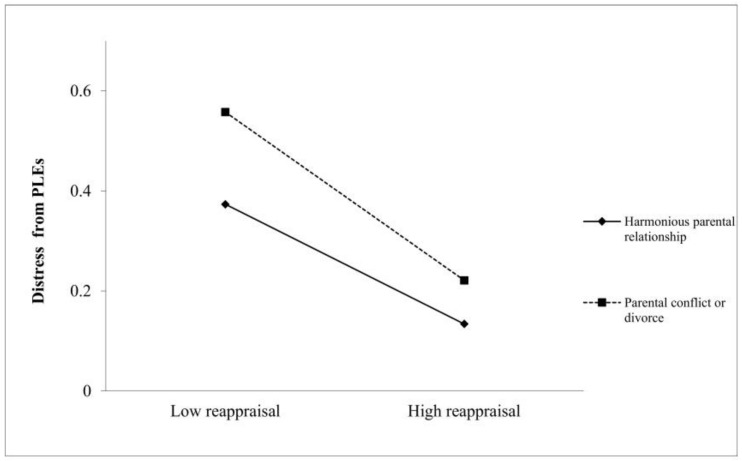
Plot of simple slopes for the interaction between parents’ relationship and the use of reappraisal on distress from PLEs.

We conducted a simple slope test to explore the interaction. As shown in Figure 1, the negative association between the use of reappraisal and distress from PLEs is stronger among those with parental conflict or divorce (βsimple = −0.027, *t* = −5.406, *p* < 0.001) than those with positive parents’ relationships (βsimple = −0.015, *t* = −6.105, *p* < 0.001). Parents’ relationship did not moderate the association between the use of suppression and distress from PLEs.

## 4. Discussion

The present study evaluated the impact of ER strategies on PLEs in the context of the parents’ relationship. Our results suggested that maladaptive ER strategy use characterized by higher use of suppression and lower use of reappraisal, as well as parental conflict or divorce, are associated with PLEs and related distress. Furthermore, parents’ relationship and ER strategy use interacted to predict distress from PLEs. The impact of lower use of reappraisal on distress from PLEs was stronger among those who had parental conflict or divorce than those with a positive parent relationship.

Our results are congruent with some previous findings of individuals with PLEs and psychosis reporting the negative influence of maladaptive emotion regulation (i.e., suppression) on psychotic symptoms [11,14,15,18]. Investigations of the relationship between reappraisal and PLEs have yielded mixed findings. Some studies reported negative or no association between reappraisal and frequency of PLEs [12,40]. In another study, Westermann et al. found that the use of reappraisal was associated with increased states of paranoia in high-paranoia-prone individuals [17]. The reasons for the inconsistency may be related to different subjects. In our study, college students may have less severe symptoms and use reappraisal more effectively. Further prospective research is needed to clarify the role of reappraisal on PLEs. Attachment theory brings a developmental perspective to the understanding of the impact of early-life affection bonds with caregivers on later emotional regulation abilities [41]. The role of attachment in psychosis is widely acknowledged. Higher rates of insecure attachment exist across the psychosis continuum [42]. People with insecure attachment styles have difficulties in emotion processing and tend to use more maladaptive ER strategies. Previous studies found that maladaptive ER can mediate the association between insecure attachment style and psychotic symptoms [43], this could be a potential explanation for the pathway from insecure attachment to psychosis. Unlike the attachment styles that primarily develop during childhood and remain stable and consistent over time, the ability to regulate emotion may be amenable to change with psychotherapy, which may be more clinically useful in prevention and intervention.

Our results are consistent with a substantial number of studies reporting the effects of negative family environment, including parental conflict and divorce, on both subclinical and clinical psychotic symptoms [27,44]. Exposure to parental conflict and divorce can be viewed as common stressful events for psychopathology in children’s developmental processes and are associated with the subsequent onset of psychotic experiences [45]. According to spill-over theory, conflicts between parents can spill over into interactions with their children [46], which may to detrimental to children’s attachment relationships with their parents. Insecure attachment due to parental conflict and divorce can negatively influence behavioral and emotional control, which may be associated with the presence of PLEs [47]. In contrast, positive parents’ relationships may be protective against poor outcomes by positive parenting and parent–child attachment [48]. However, the onset and duration of conflict within marriage and how long the conflict ends after divorce may also account for the different outcomes of young people. Further research is needed to clarify these relationships.

This study provides the first evidence that a maladaptive pattern of ER strategy was associated with the distress of PLEs depending on individuals’ parents’ relationships. Specifically, positive parents’ relationships could alleviate the adverse effect of maladaptive ER strategy use, while higher parental conflict and divorce may exacerbate the distress of PLE symptoms amongst those with emotion-regulation difficulties. One possible explanation is that maladaptive ER strategies may lead to difficulties in dealing with negative emotions resulting from family stress for children from high parental–conflict homes and divorced families. Yee et al. found that individuals at clinically high risk for psychosis tended to use coping strategies closely associated with depression and anxiety in response to family stress [44]. Children’s cognitive appraisals and affective responses to parental conflict were associated with their maladjustment [49,50,51]. In our study, the moderating effect only existed for PLE-related distress. Distress from subclinical psychotic symptoms is not necessarily associated with frequency of symptoms but is a predictor of symptom severity over time [52]. It may be speculated that this moderate effect is more likely associated with the severity of symptoms, although a follow-up study is needed to clarify this link. Although this may not imply diagnostic specificity, previous studies showed that PLE-related distress was associated with increased risk of transition to psychosis, help-seeking behavior, functional impairment, other reported psychopathology, delayed developmental milestones, and altered structural MRI metrics [53,54,55,56], and the negative impact of distress was greater in those with persistent PLEs [56]. The role of distress from symptoms remains unclear and requires further investigation. In this study, only reappraisal interacted with parental conflict in the distress from PLEs, indicating that reappraisal may be a more efficient ER strategy against distress from PLEs in those with negative parents’ relationships. Our findings may have implications for intervention for young people with PLEs. Psychotherapy emphasizing a greater focus on reappraisal rather than suppression and combined with family intervention to reduce the adverse influence of the negative parents’ relationship could be helpful.

There are several limitations to our study. The causal relationships between the parents’ relationship, ER strategies, and PLEs cannot be determined due to the cross-sectional design. This study used a self-report tool to measure PLEs due to the high false-positive rate of PLEs reported in the general population. One problem is that the sample in this study was comprised of adolescents, who may not be able to reliably answer these questions. Therefore, it is necessary to control the quality of the responses, such as through interviews assessed by objective raters and including indexes of severity, pervasiveness, frequency, sincerity, social desirability, conviction of symptoms, etc. [57]. For self-report scales, in addition to the use of the subscales and checking the rationality of the responses, similar psychometric properties to findings from clinical studies can serve as indicators of diagnostic validity [58,59]. Another limitation is that our study did not include clinical samples to test whether our hypothesis applies specifically across the psychosis continuum rather than only to PLEs, which are generally associated with psychopathology. As mentioned by Ising et al., the PQ-16 only measures the present and related distress of PLEs, and the intensity of symptoms should be added to improve the sensitivity to distinguish whether such experiences are associated with a high risk status or clinical psychosis [36]. The current findings cannot be directly generalized to clinical samples. Moreover, the parents’ relationship was measured based on self-report surveys to investigate whether parents were highly conflicted or divorced at only one time point, making it impossible for our study to clarify the complexity of parental conflicts and divorces. Previous studies suggest that the age of exposure to parental conflict and the temporal dynamics of parental conflict are associated with youth adjustment [60]. Using dichotomous variables means that some details about parents’ relationships are lost, a detailed definition of the parents’ relationship should include categories such as whether they were recently divorced, not divorced but in longstanding conflict, or divorced but reconciled, and further longitudinal studies should also include variables such as the exposure age to parental conflict or divorce, the duration and temporal dynamics of parental conflicts during the marriage or before divorce, and the family structure after divorce to fully capture the processes relating the parents’ relationship to PLEs. Finally, previous studies suggested that the impact of parental conflict on children depends on children’s attributions and emotional processing of the conflict [29]. In this study, a focus on adolescent perception of the parents’ relationship can capture the effects of the spill-over of family dysfunction on adolescent emotional functioning. Further research adding multi-source information on the parents’ relationship may provide a larger perspective framework for understanding the links between the parents’ relationship and children’s ER and PLEs.

In conclusion, our findings agreed with and extended previous research by showing the direct and interactive contribution of the parents’ relationship and ER strategies in the presence of PLEs and PLE-related distress among adolescents. Less use of reappraisal seems to be related to more distress from PLEs for those with parental conflict or divorce, while positive parental relationships buffer against the adverse effects of a maladaptive pattern of ER strategies on distress from PLEs. Adolescence is a period that could be vulnerable to family influence, the emergence of psychosis, and emotional dysregulation. Our results have the potential to inform those in public health and clinical practice about the parents’ relationship and emotion regulation may serve as useful information for the risk assessment and prevention of PLEs or psychotic symptoms in non-clinical adolescents and potential targets for early interventions for help-seeking individuals.

## Figures and Tables

**Table 1:** Sample characteristics.

Variables	Mean (SD) or *n* (%)
Age, Mean (SD)	17.9 (0.7)
Gender, male, *n* (%)	1659 (61.3)
Single-child, *n* (%)	1910 (70.5)
Family history of mental disorders, *n* (%)	122 (4.5)
Monthly family income (CNY), *n* (%)	
<2000	191 (7.1)
2000–4999	717 (26.5)
≥5000	1800 (66.5)
Parents’ relationship, *n* (%)	
Harmonious	2053 (75.8)
Conflicting or divorced	655 (24.2)
PQ-16 Items endorsed	4.18 (3.20)
PQ-16 Distress	1.32 (2.82)
ERQ Reappraisal	30.59 (5.56)
ERQ Suppression	16.21 (4.60)

**Table 2:** Regression for psychotic-like experiences (PLEs) on emotion-regulation strategies, parents’ relationships, and their interaction effects.

	PQ-16 Items Endorsed		PQ-16 Distress	
	β	95% CI	β	95% CI
Reappraisal	−0.06	−0.10, −0.02 **	−0.12	−0.16, −0.08 **
Suppression	0.32	0.28, 0.37 **	0.25	0.21, 0.29 **
Parents’ relationship	0.10	0.07, 0.14 **	0.16	0.12, 0.20 **
Reappraisal × Parents’ relationship	0.01	−0.05, 0.04	−0.05	−0.09, −0.01 *
Suppression × Parents’ relationship	−0.02	−0.06, 0.02	0.03	−0.02, 0.06
R^2^	0.123		0.138	
*P*	<0.001		<0.001	

Age, sex, and single-child status were controlled for. β, standardized regression coefficient; * *p* < 0.05, ** *p* < 0.01.

## Data Availability

The data presented in this study are available on request from the corresponding authors.
